# Ambient air pollution and adverse birth outcomes: A review of underlying mechanisms

**DOI:** 10.1111/1471-0528.17727

**Published:** 2023-11-30

**Authors:** Julia C. Fussell, Eric Jauniaux, Rachel B. Smith, Graham J. Burton

**Affiliations:** 1 MRC Centre for Environment and Health, School of Public Health, Imperial College London, London, United Kingdom; 2 National Institute for Health and Care Research Health Protection Research Unit in Environmental Exposures and Health, Imperial College London, London, United Kingdom; 3 EGA Institute for Women's Health, Faculty of Population Health Sciences, University College London, London, UK; 4 Mohn Centre for Children’s Health and Wellbeing, School of Public Health, Imperial College London, London, UK; 5 Department of Physiology, Development and Neuroscience, University of Cambridge

**Keywords:** adverse birth outcomes, ambient air pollution, endocrine disruption, epigenetic changes, inflammation, mechanisms, oxidative stress, placenta

## Abstract

Epidemiological data provide varying degrees of evidence for associations between prenatal exposure to ambient air pollutants and adverse birth outcomes (suboptimal measures of fetal growth, preterm birth and stillbirth). To further assess certainty of effects, this review examines the experimental literature base to identify mechanisms by which air pollution (particulate matter, nitrogen dioxide and ozone) could cause adverse effects on the developing fetus. It likely that this environmental insult impacts multiple biological pathways important for sustaining a healthy pregnancy, depending upon the composition of the pollutant mixture and the exposure window owing to changes in physiologic maturity of the placenta, its circulations and the fetus as pregnancy ensues. The current body of evidence indicates that the placenta is a target tissue, impacted by a variety of critical processes including nitrosative/oxidative stress, inflammation, endocrine disruption, epigenetic changes, as well as vascular dysregulation of the maternal-fetal unit. All of the above can disturb placental function and, as a consequence, could contribute to compromised fetal growth as well increasing the risk of stillbirth. Furthermore, given that there is often an increased inflammatory response associated with preterm labour, inflammation is a plausible mechanism mediating the effects of air pollution on premature delivery. In the light of increased urbanisation and an ever-changing climate, both of which increase ambient air pollution and negatively affect vulnerable populations such as pregnant individuals, it is hoped that the collective evidence may contribute to decisions taken to strengthen air quality policies, reductions in exposure to air pollution and subsequent improvements in the health of those not yet born.

## 1 Introduction

Air pollution has been defined by the World Health Organization (WHO) as the contamination of the indoor or outdoor environment by any chemical, physical or biological agent that modifies the natural characteristics of the atmosphere^1^. Findings from epidemiological and toxicological research have also confirmed that this heterogeneous mix of gases and particulate matter (PM) modifies public health^2^. A convincing body of evidence exists linking exposure to ambient air pollutants and cardiorespiratory disease^3–6^. Data from an increasing number of studies also suggest this environmental threat contributes to a broader number of health effects including diabetes^7^, suboptimal cognitive development^8^, cognitive decline^9^ and adverse birth outcomes including preterm delivery^10^, low birth weight (LBW)^11^ and stillbirth^12^. Most of these complications have also been associated with maternal exposure to tobacco smoke and are likely to be triggered by similar mechanisms i.e. inflammation and oxidative stress^13–16^. It is also likely that air pollutants can induce similar placental epigenetic alterations as maternal smoking^17–19^, highlighting the possible long-term effects of in-utero exposure on the susceptibility of childhood and adult diseases. This review stems from a larger ongoing piece of work being undertaken by the UK government’s Committee on the Medical Effects of Air Pollutants on adverse birth outcomes^20^. After a brief overview of the epidemiological evidence base, it summarises, through the discussion of studies that have produced results of special interest, our current understanding of the mechanisms activated in placental tissue through which exposure to particulate matter, nitrogen dioxide and ozone (i.e. the modern day air pollutants that are most [a] abundant in today’s urban environments and [b] widely studied for their health impact) may contribute to adverse birth outcomes.

## 2 Epidemiological Evidence Of The Effects Of Air Pollution On Adverse Birth Outcomes

The epidemiological literature base of studies, systematic reviews and meta-analyses investigating associations between maternal exposure to air pollutants and birth outcomes is rapidly expanding. It is also characterised by inconsistency (i.e. reporting positive and null associations) in the collective evidence. The most recent authoritative reports by professional bodies on ambient pollutants are the US Environmental Protection Agency Integrated Science Assessments (ISAs)^21–23^. The ISA for PM concluded that many studies provide evidence for a positive association between particulate matter less than 2.5 μm in diameter (PM_2.5_) exposure and fetal growth and PTB^21^. The results of a recent large meta-analysis of ambient PM_2.5_ during entire pregnancy and adverse birth outcomes build upon these conclusions, reporting summary estimates of 22 grams lower birth weight (95% uncertainty intervals [UI] 12, 32; estimates from 44 studies), 11% higher risk of LBW (1.11 95% UI 1.07, 1.16; 40 studies) and 12% higher risk of PTB (1.12 95% UI 1.06, 1.19; 40 studies), each per 10 μg/m^3^ increase in ambient PM_2.5_^24^. It is noteworthy that in another meta-analysis, negative associations between a change in grams (β) of term birth weight and ambient PM_2.5_ across the entire pregnancy were observed at concentrations lower than 10 μg/m^3^ (the WHO air quality guideline value)^25^. The pooled estimates per 10 μg/m^3^ increment in PM_2.5_ were β=−15.58 g (95% CI −25.38, −5.79) in the low PM_2.5_ exposure subgroup (<10 μg/m^3^; 6 studies) and β=−16.58 g (95% CI −20.35, −12.81) in the high PM_2.5_ exposure subgroup (≥10 μg/m^3^; 20 studies).

The ISA for NOx reports that there is largely consistent evidence to support an association between exposure to NO_2_ and fetal growth restriction (FGR) but inconsistent results from studies that examined birth weight outcomes and PTB^22^. A recent meta-analysis did not find an association between entire pregnancy NO_2_ exposure and PTB (RR 1.010 95% CI: 0.990, 1.030 per 10 μg/m^3^ NO_2_; 20 studies) reflecting inconsistency in direction of associations observed in underlying studies^26^. The same meta-analysis reported an inverse association for first trimester NO_2_ (RR 0.972 95% CI: 0.950, 0.994 per 10 μg/m^3^ NO_2_; 21 studies), a null finding for second trimester NO_2_ (RR 1.002 95% CI: 0.970, 1.034 per 10 μg/m^3^ NO_2_; 18 studies) and a positive association for third trimester NO_2_ exposure and PTB (RR 1.066 95% CI: 1.031, 1.102 per 10 μg/m^3^ NO_2_; 15 studies). The ISA for O_3_ concludes that whilst there is some evidence for the effects of O_3_ on fetal growth (especially for continuous term birth weight), findings were more consistent for an elevated risk of PTB with ozone exposure during early to mid pregnancy^23^. A recent meta-analysis focusing on early pregnancy exposures is supportive of the latter^27^. The pooled odds ratio (OR; 95% CI) for a 10 ppb increase in ozone exposure was 1.06 (1.03, 1.10) in trimester 1 (17 studies) and 1.05 (1.02, 1.08) in trimester 2 (15 studies).

The epidemiological evidence base for ambient air pollution and stillbirth remains limited. Conclusions in the ISAs included the presence of generally positive associations for PM_2.5_^21^ and inconsistent findings for O_3_^23^. A recent meta-analysis also reported positive associations with risk of stillbirth for entire pregnancy PM_2.5_ (OR 1.10 95% CI 1.07, 1.13; 7 studies), third trimester PM_2.5_ (1.09 95% CI 1.01, 1.18; 5 studies), and first trimester O_3_ (OR 1.03 95% CI 1.00, 1.05; 6 studies), each per 10 μg/m^3^ increment, but no associations for NO_2_^12^. The effects of traffic-related air pollutants, which dominate exposures in urban environments where the majority of people live, work and commute, on adverse birth outcomes (term LBW, SGA, term birth weight, PTB) have also undergone a recent evaluation by the Health Effects Institute^28^. For traffic-related PM_2.5_, the meta-analytical summary relative risks per 5 μg/m^3^ increment were 1.11 (95% CI: 1.03; 1.20) for term LBW, 1.09 (1.04; 1.14) for SGA and a mean difference in term birth weight of -17.3 (-33.2; -1.5) grams. In contrast with the findings for ambient PM_2.5_ in general, the summary estimate for traffic-related PM_2.5_ and PTB was null (RR 0.99 (95% CI: 0.90–1.09)), likely reflecting compositional differences in the particulate mix. Apart from an association between NO_X_ and term low birth weight (RR 1.02 (1.01–1.03) per 20 μg/m^3^ increment)), results for traffic-related NO_2_, NO_X_ or elemental carbon in relation to risk of term LBW, continuous birth weight at term, SGA or PTB were largely null.

## 3 General Mechanisms Of Toxicity Of Air Pollutants

A succession of events involving gaseous and particulate pollutant-induced pulmonary and systemic oxidative stress and inflammation, mediated via redox sensitive signaling pathways, are considered integrative biological pathways underlying the harmful effects of ambient pollution on the cardiorespiratory system^29, 30^. An excessive oxidative challenge, as opposed to the degree that is essential in physiological redox signaling and for steady-state maintenance of stress response systems, creates an imbalance between the generation of reactive oxygen species (ROS) and antioxidant protection in favour of the former, causing excessive oxidative damage^31^. Reactive nitrogen species (RNS) also exist and when overproduced or under eliminated can create damage via nitrosative stress. Biomolecules that are targeted by ROS/RNS include DNA, proteins, lipids, and carbohydrates, giving rise to DNA mutations, lipid peroxidation and protein oxidations respectively. This can lead to appreciable impairment of cellular function and in the worst case to cell death, organ dysfunction and disease^31^.

Pollutants that make up the ambient pollutant mix are free radicals (e.g. NO_2_) or have the ability to drive oxidative reactions (e.g. PM, O_3_). Once inhaled, O_3_ and NO_2_ react with proteins and lipids present in the lung lining fluid compartment to produce secondary oxidant species^32^. This means that cellular responses to concentrations of gaseous pollutants that overcome endogenous antioxidant defences are not the result of a direct reaction of the pollutant with epithelial cell surface components, but instead, are mediated via a cascade of secondary, free radical derived products. Several more complicated and inter-related pathways exist by which inhaled PM can generate oxidative stress at the air-lung interface. These include an ability of the particle surface per se to elicit oxidative stress and the carriage of direct redox catalysts (e.g. transition metals, quinones) or compounds (e.g. polycyclic aromatic hydrocarbons [PAHs]) that can be metabolized to reactive electrophiles in vivo^33^.

On reaching the lung surface, secondary oxidation products arising from gaseous and particulate pollutants, initiate (via the activation of transcription factors) cytokine and chemokine generation, adhesion molecule expression and tight junction modification. These responses lead to the influx of activated inflammatory cells from the vasculature into the lung, which generates a second wave of pulmonary oxidative stress accompanied by systemic inflammation and oxidative imbalances. The latter, plus the translocation of ultrafine and nanosize particles and/or particle constituents (organic compounds, metals) across the alveolar membrane into the general circulation paves the way for toxicity within the vasculature and at organ sites distant from the lung, including the placenta^34^.

## 4 Mechanisms Underlying Adverse Birth Outcomes

The convincing evidence that air pollution-induced oxidative stress and inflammation play critically important roles in cardiorespiratory diseases, including maternal cardiovascular health^35^, suggests that the epidemiological associations observed between poor air quality and adverse birth outcomes could be mediated, in part, by these key pathways. Air pollution, however, likely impacts multiple biological mechanisms important for sustaining a healthy pregnancy, depending upon the composition of the pollutant mixture, and the exposure window owing to changes in physiologic maturity of the placenta, circulations and fetus as pregnancy ensues.

### 4.1 Human studies

The experimental research summarised below originates from human pregnancy cohort studies that have assessed, for example, relevant biomarkers in biological samples (i.e. maternal blood and urine, placental tissue, cord blood) collected during pregnancy and at birth. These studies are vital to further our understanding, owing to specific inadequacies (e.g. fundamental differences in gestational sac structure, placentation, circulations, fetal/placental weight ratios, organogenesis phases and gestational length) of animal models. Whilst Table S1 provides details those studies linking maternal exposure to ambient air pollutants to a given mechanistic endpoint and an adverse birth outcome, Table 1 provides a top-level summary of the mechanistic changes that have been observed.

#### 4.1.1 Effect on the placental circulations

The oxidative and inflammatory properties of air pollution are linked to many pathophysiological changes in the cardiovascular system that ultimately increase cardiovascular morbidity and mortality. These include prominent effects on the vasculature leading to endothelial dysfunction and arterial vasoconstriction^36^ which are both associated with PTB and abnormal fetal growth^37^.

Studies have investigated placental vascularization and function in relation to air pollution exposure and have reported contradictory effects on placental vascular resistance to blood flow in the uterine circulation^38–43^. Two of these studies, using maternal personal exposure, found that higher NO_2_ exposure^40, 43^ is associated with a lower mean artery pulsatility index (PI). Conversely, other studies reported that maternal exposure to NO_2_ is associated with an increased uterine resistance index (RI)^39^ and increased incidence of bilateral uterine notching^42^. Similarly, Ouidir et al (2021) observed an increased resistance index of the left uterine artery associated with exposure to NO_2_ as well as PM_2.5_^41^.

#### 4.1.2 Oxidative stress

Human placentation is characterised by the migration of placental trophoblastic cells into the uterine wall, transforming the maternal arteries into low-velocity, high-conductance vessels and controlling the entry of oxygenated maternal blood to the placenta^44, 45^. Disruption of this process is associated with hyperoxia or fluctuating levels of oxygen inside the placenta – disturbances found in common pregnancy complications such as miscarriage, preeclampsia and FGR^46, 47^. Oxygen free radicals are an inevitable by-product of aerobic metabolism and some degree of placental oxidative stress likely occurs at the end of the first trimester^48^, regulating formation of the placental membranes^49^. Placental malperfusion generates chronically high levels of oxidative stress causing indiscriminate damage to biomolecules and disruption of signalling pathways. These have immediate consequences for the outcome of a pregnancy, but also predispose the offspring to metabolic, cardiovascular, and neuropsychiatric disorders and certain cancers in adult life due to structural and epigenetic changes in organ systems^50^.

Exacerbation by maternal exposure to air pollution of the heightened oxidative state during a standard pregnancy may overcome mechanisms that minimise the deleterious effects of ROS production. Several approaches have been undertaken to investigate an exacerbation of oxidative stress in response to ambient air pollution exposure during gestation and whether such an effect has the potential to adversely influence birth outcomes. These include studies on biomarkers of nitrosative/oxidative stress, oxidative potential (OP; the capacity of particles to directly cause damaging oxidative reactions) of ambient pollution and genetic susceptibility due to polymorphisms in antioxidant genes.

Biomarker studies have shown associations between ambient concentrations of PM and NO_2_ and markers of nitrosative (3-nitrotyrosine [3-NT]) and oxidative (8-oxo-7,8-dihydro-2’-deoxyguanosine [8-OHdG]) stress in the placenta and/or maternal/cord blood ^51–54^ (Table 1). 3-NT is the stable product of tyrosine nitration with the reactive oxygen species peroxynitrite and whilst nitration of placental proteins is evident in standard pregnancies, the presence of nitrosative stress has also been linked with diminished placental vascular reactivity^55^ that in turn, could compromise placental function and thus fetal development and growth.

A number of studies have also observed significant associations between increased concentrations of PM^56–58^ and NO_2_^59^ during pregnancy and decreased placental/cord blood mtDNA content (Table 1), indicative of mitophagy and mitochondrial death^60^. By regulating energy, placental mitochondria are essential in the proper formation and functioning of the organ and a healthy pregnancy^61^. Mitochondria are the major intracellular source and primary target of ROS and, compared to nuclear DNA, mtDNA is more sensitive to oxidative stress due a lack of protective chromatin structure, histones, and introns and less efficient repair mechanisms^62, 63^. Oxidative stress therefore has the potential to adversely affect placental mitochondria and in doing so impair the ability of the placenta to support the growing fetus through energy-dependent processes such as active transport and hormone secretion. Estimates from one study indicated that a 10 μg/m^3^ increment in average NO_2_ exposure during pregnancy was associated with a 4.9% decrease in placental mtDNA content and a 48g decrease in birth weight^59^ (Table S1). However other studies examining links between air pollution concentrations, markers of oxidative stress and an adverse birth outcome do not however provide evidence that an increased oxidative burden with increased exposure during pregnancy is associated with preterm or negative indices of fetal growth^52–54^. Studies that have sought to evaluate the involvement of oxidative stress by examining whether the OP of PM_2.5_ modifies the relationship between PM_2.5_ mass concentrations/exposures and adverse birth outcomes have reported mixed results, possibly indicative of different assays employed and birth outcomes evaluated^64, 65^ (Table S1). Studies focusing on gene-environment interactions in pregnant women suggest an interaction between exposure to air pollution during pregnancy and genetic polymorphisms in antioxidant genes that results in an increased risk of preterm delivery^66, 67^ (Table S1).

#### 4.1.3 Inflammation

Maternal inflammatory responses are modified to establish and maintain a viable pregnancy. Whilst the second trimester is a predominantly anti-inflammatory state, elevated inflammatory signals take place during implantation^68^, placentation^69^ and in preparation for delivery^70, 71^. It is possible therefore that the proinflammatory effects of air pollutants could disturb these delicate balances and in doing so elicit adverse birth outcomes. In this regard, PTB is generally regarded as a syndrome resulting from multiple causes including inflammation^72^ through the early activation of cytokines such as interleukin (IL)-1β and tumour necrosis factor-α (TNF-α), which are otherwise part of the body's normal preparatory step for term deliveries^73, 74^. Furthermore, inflammation may affect placental growth, development and function, which in turn can lead to FGR^75^, whilst cytokines can cross the placental barrier and interfere with fetal development^76^.

Studies investigating whether air pollution impacts the maternal-fetal inflammatory response, measuring intrauterine inflammation or biomarkers in maternal blood in early/mid pregnancy and/or cord blood, have reported associations with ambient concentrations of PM^77–80^ and O_3_^77, 80^ (Table 1). Whilst concentrations of NO_2_ have not been associated with a maternal inflammatory response, associations with higher cord blood CRP concentrations have been reported^77, 78^ (Table 1). Only one of these studies extended their analysis to look for associations between the maternal (but not cord blood) inflammatory biomarkers and birth outcomes^80^ (Table S1). No associations were observed for birth weight, whilst elevated levels of maternal CRP were associated with modestly older gestational age at birth. As a result of the inflammatory response to pregnancy, maternal CRP levels increase slightly during standard pregnancies, generally peaking during the third trimester^81, 82^. However, a greater increase in CRP levels has been reported in women whose pregnancies are complicated by FGR and preterm delivery^83–85^. Furthermore, elevated CRP levels in cord blood have been found in SGA neonates^86^.

PM_2.5_ exposures in the first trimester or around mid-gestation is associated with an increase in maternal serum IL-1β, IL-6 and TNF-α^87^. PM_10_ exposure has also been positively associated with serum cytokines IL-6 but inversely associated with cervico-vaginal cytokine TNFα^88^.

#### 4.1.4 Epigenetic alterations

High DNA synthesis rates and extensive epigenetic remodeling (e.g. methylation, demethylation and re-methylation) during embryogenesis^89, 90^ suggest that the fetal epigenome is a link between early life exposure to environmental factors and both adverse birth outcomes and later life events^91–93^. Epigenetic regulation of genes is also crucial in placental growth and functioning^94^. Modification, during a susceptible time window, of expression of genes involved in key placental cellular processes has the potential to contribute to abnormal placental and/or fetal development. Indeed, alterations in placental DNA methylation patterns and microRNA expression have been reported in association with fetal growth and adverse maternal exposures such as alcohol and tobacco smoke^95, 96^.

Studies exploring whether prenatal exposure to air pollution is associated with epigenetic modifications and adverse birth outcomes have primarily focused on global DNA methylation or differential gene-specific methylation in placental tissue or maternal/cord blood at birth. Those that have examined global methylation or DNA methylation within LINE1 repetitive elements (frequently used as surrogate markers for global methylation) have observed a lower degree of methylation in association with exposure to PM^97–99^ and living close to a major roadway^100^ (Table 1). Decreased DNA methylation has also been observed in the few studies that looked at associations with NO_2_ and O_3_^101^.

The finding of a lower degree of placental global DNA methylation in association with exposure to ambient PM in early pregnancy^99^, including the critical stages of implantation, is of interest considering that disturbance of maintenance DNA methylation in placental tissue is associated with abnormal embryonic development in the mouse model^102^. Moreover, LINE-1 down-regulation can induce an inflammatory response, potentially explaining the negative correlations that have been reported with PTB^103^. Studies focusing on locus specific methylation (and importantly genes relevant to placental/fetal development), have found that prenatal exposure to particulate or gaseous pollutants are associated with altered DNA methylation of *HSD11B2* (glucocorticoid metabolism)^98^ and *H19* (fetal growth)^104^ (Table 1). The negative associations between LEP methylation status in the placenta and both PM_2.5_ exposure and placental NTp, suggest that oxidative/nitrosative stress might contribute to associations between PM_2.5_ and placental epigenetic events. Indeed, one mechanism by which air pollution exposure is thought to modify DNA methylation involves the reaction of ROS and RNS with DNA with subsequent strand breaks, base modification, and inter-strand and intra-strand crosslinks. Global hypomethylation ensues, owing to the inability of DNA methyltransferases to recognize the damaged DNA^17^.

Little evidence exists however that these epigenetic changes may be an underlying mechanism by which air pollution may adversely affect birth outcomes. The majority of studies have not extended their analysis to look at a mediating effect of disrupted DNA methylation patterns on the relationship between air pollution exposure during pregnancy and adverse birth outcomes or found no significant associations between an epigenetic modification and an adverse birth outcome (Table S1). Cai et al (2017) however reported that associations of PM_10_ exposure during early pregnancy with DNA methylation were more evident in FGR newborns compared to normal newborns^98^, whilst Vos et al (2020) reported a significant association between mitochondrial DNA methylation and birth weight^105^.

#### 4.1.5 Endocrine disruption

A steady balance of thyroid hormones regulate metabolism and stimulate differentiation and growth of the fetus. During the first trimester the fetus depends on a maternal supply of thyroid hormones until the thyroid gland becomes fully functional at around 10-12 weeks of gestation^106^. Maternal hyper- and hypothyroidism are associated with increased risk of a LBW^107, 108^ and studies also suggest that fetal thyroid function is instrumental in regulating fetal growth^109, 110^. A number of studies have suggested that the maternal^111–114^ and fetal^111, 115, 116^ thyroid glands may be susceptible to prenatal PM exposures. Findings from Janssen et al (2017) highlight the potential influence of PM_2.5_ exposure on fetal thyroid function and fetal growth^111^. Ambient PM_2.5_ concentrations were inversely associated with cord blood thyroid stimulating hormone (TSH) and free thyroxine (FT4) concentrations and the free thyroxine/free triiodothyronine ratio (FT4/FT3) ratio and positively associated with FT3 levels. Although neither FT3 nor TSH levels in maternal or cord blood were associated with birth weight, an 11% decrease in cord blood FT4 was associated with a 56 g decrease in in mean birth weight (Table S1). Shields et al. (2011) reported that in healthy pregnancies, placental weight was positively associated with cord blood FT4 levels and posited that thyroid hormones could influence fetal growth indirectly by affecting placental growth^110^. These researchers also reported that lower FT4 levels in cord blood were associated with a lower birth weight. A role for oxidative stress in the disruption of thyroid function by PM is supported by findings of increased ROS concentrations in goitrous thyroid glands^117^.

An effect of maternal exposure to PM_10_ and NO_2_ on placental development and function is supported by one study reporting associations with lower concentrations of placental growth factor (PlGF) and higher concentrations of soluble fms-like tyrosine kinase 1 (sFlt) in umbilical cord blood^42^ (Table S1). Whilst PlGF is important for placental development and angiogenesis, sFlt-1 binds to this protein and thereby inhibits its activity ^118^. The findings of van den Hooven et al (2012) are consistent with an anti-angiogenic state. PM_10_ and NO_2_ exposures were not associated with placenta to birth weight ratio but were associated with lower placenta weight.

#### 4.1.6 Insights from metabolomic research

Metabolomic research is being increasingly used in maternal-fetal medicine to identify biological changes associated with fetal growth^119^. Examples include the analysis of maternal plasma and urine to identify metabolites predictive of small for gestational age babies and a PTB^120–122^. For studies investigating metabolic changes in response to environmental exposures, analysis of amniotic fluid, the placenta and cord blood would be particularly relevant as the sample contains the essential nutrients, hormones, and immunological factors as well as potentially harmful xenobiotic metabolites to which the developing fetus is directly exposed. The use of metabolomics to identify altered biological pathways in the fetus in response to prenatal exposure to air pollution is a recent area of research^123–125^. It is noteworthy however that among the metabolomic features and pathways identified to date, are oxidative stress and inflammation, i.e. mechanisms already implicated using more traditional approaches^124^. A study by Laine et al (2020) assessing the effects of prenatal exposure to mixtures of air pollutants (PM_2.5_, PM_10_, NO_2_, NO_x_, ultrafine particles and OP of PM_2.5_) on birth weight in a pooled birth cohort assessed mechanistic interactions between metabolomic signatures and targeted inflammatory proteins in cord blood pooled^125^ (Table S1) Exposure to 3 different mixture pollutant models was found to be negatively associated with birth weight. For example, increased concentrations of a PM_2.5_, PM_10_, and NO_2_ mix was estimated to result in an approximate 96 g decrease in birth weight. This relationship was found to be mediated by inflammatory proteins (IL-17 and epidermal growth factor) and 665 metabolomic features, including several involved in fetal development and growth.

### 4.2 Animal studies

Whilst recognizing that animal studies are less useful for understanding the underlying effects of air pollution on adverse birth outcomes in humans (e.g. owing to distinct differences in human and rodent placentas), experimental studies do have value given that so much of the human mechanistic data is associative. Well-characterised exposures and the capacity for invasive procedures are strengths of animal inhalation experiments that facilitate the demonstration of biological plausibility for effects through various biological mechanisms. Studies in rodents investigating effects of maternal exposure to diesel exhaust (DE) or DE particles (≥ 300 μg PM/m^3^ before and/or during gestation) have reported several effects on placentation including decreased vascularisation and perfusion^126^, injury in association with increased embryo resorption^127^ and a reduction in cells responsible for structural integrity^128^. Pregestational and gestational exposure of mice to ambient air (PM_2.5_: 27.5 μg/m^3^; NO_2_: 101 μg/m^3^; CO: 1.81 μg/m^3^; SO_2_: 6.66 ppm) in exposure chambers placed in a location with high traffic density resulted in decreased fetal weights and changes in functional morphology of the placenta^129^ and umbilical cord^130^. Exposure of rats before (5 times per week for 3 weeks) and/or during pregnancy (for 14 days) to concentrated PM_2.5_ (600 μg/m^3^ for 60 minutes) lowers placental concentrations of the angiogenic factors (vascular endothelial growth factor and its receptor fetal liver kinase 1)^131^. A number of these changes were accompanied by an inflammatory response and oxidative stress^127, 128, 130, 132, 133^. Ozone exposure (0.4 or 0.8 ppm 4 hours/day during implantation) led to changes in uterine arterial resistance and a lower offspring weight^134, 135^.

### 4.3 In vitro studies

A number of in vitro studies have been performed to explore mechanistic insights into how air pollution may affect placental structure and function. Most of these have been based on HTR-8/SVneo cells, a widely used first-trimester trophoblast cell line but that has been shown to be a mix of trophoblast and mesenchymal cells^136^. Caution should therefore be exercised when extrapolating results based on these cells to the placenta. Nonetheless, culture of HTR-8/SVneo cells with either PM_2.5_ or PM_10_ collected at sites of urban traffic showed uptake of particles into perinuclear endosomes within 30 minutes of exposure^137^ and the mitochondria by 24 hours in association with vacuolation of organelles, dilation of endoplasmic reticulum cisternae and chromatin aggregation within the nucleus^138^. High doses of urban PM_2.5_ or PM_10_ (5000 ng/ml) caused increased secretion of IL-6 and reduced secretion of human chorionic gonadotropin by 48 hours, whereas release of progesterone was either increased or not affected^137, 138^. Proteomic analysis revealed 29 differentially expressed proteins consistent with the pattern observed in cases of FGR. Cell number was decreased after 7 days, due to increased cell death^138^. Overall, the findings suggest PM induced activation of stress pathways seen in the placenta in cases of FGR^139^. These results are consistent with findings of cytotoxicity and reduced proliferation of HTR-8/SVneo cells following exposure to PM_2.5_, with cell cycle arrest at the G2/M phase^140^. In addition, a reduced mitochondrial membrane potential consistent with induction of apoptosis was demonstrated. Importantly for placentation, the invasive potential of the cells was significantly reduced due to increased expression of the migration inhibitors TIMP1 and TIMP2. Deficient, shallow trophoblast invasion is a unifying feature of many complications of pregnancy^141^.

## 5 Conclusions

Epidemiological data provide varying degrees of evidence, albeit inconsistent at times, for associations between concentrations of ambient air pollutants during pregnancy and adverse birth outcomes. Overall, the effects of air pollution may represent a milder version of what has been reported for active maternal smoking that is strongly linked to premature delivery and FGR^13^. Within this context, one of the main biases of large epidemiological studies is the lack of accurate data on both maternal active and passive smoking. Exposure to ambient air pollution and tobacco smoke during pregnancy are likely to have a cumulative effect on the incidence of PTB and SGA.

To further assess the certainty of effects stemming from poor ambient air quality, biological plausibility can be sought, by identifying the mechanisms, by which exposure to air pollution could cause adverse effects on the developing fetus. Although the underlying mechanistic pathways are yet not elucidated, the current body of experimental evidence does lend biological plausibility, indicating that the placenta is a target tissue, impacted by a variety of critical processes including nitrosative/oxidative stress, inflammation, endocrine disruption, epigenetic changes, as well as vascular dysregulation of the maternal-fetal unit. All of the above can disturb placental function and, as a consequence, could contribute to compromised fetal growth as well increasing the risk of stillbirth. Furthermore, given that there is an increased inflammatory response during preterm delivery, inflammation is a plausible mechanism mediating the effects of air pollution on premature labour.

Caution should be taken in the interpretation of the findings from the human mechanistic studies, partly owing to the associative nature of much of the evidence as well as some notable limitations. Since not all studies linked concentrations of air pollutants and a given mechanistic endpoint with a clinical outcome, the relevance of experimental findings to the epidemiological evidence may be questioned. Furthermore, whilst there has been an emphasis on ambient PM, effects of gaseous pollutants are under researched. The potential for misclassification of exposure is also high since the majority of the epidemiological studies with a nested mechanistic component use measures of air pollution exposure generated from regional monitoring stations and modeling methods. Such studies do not allow granular measurement of changing exposure when study participants travel to different (in/outdoor) microenvironments. It is important therefore that animal studies report adverse effects on placentation and fetal weight and are supportive of a contribution by oxidative and inflammatory pathways and that in vitro work suggests pollutants can induce activation of stress pathways seen in the placenta in cases of FGR. Future studies however should take into account the sex of the fetus since mounting evidence indicates that the placenta responds differentially to stress in a sex-dependent fashion^142^.

In summary, the current body of experimental evidence demonstrates that ambient air pollutants potentially affect a variety of critical processes that may underpin adverse birth outcomes caused by poor air quality during pregnancy. In doing so, they begin to add support for causality of epidemiological associations. In the light of increased urbanisation and an ever-changing climate, both of which increase ambient air pollution and negatively affect vulnerable populations such as pregnant individuals, it hoped that the collective evidence may contribute to decisions taken to broaden air quality policies, reductions in exposure to air pollution and subsequent improvements in the health of those not yet born.

## Supplementary Material

Supplementary Material

## Figures and Tables

**Figure 1:**
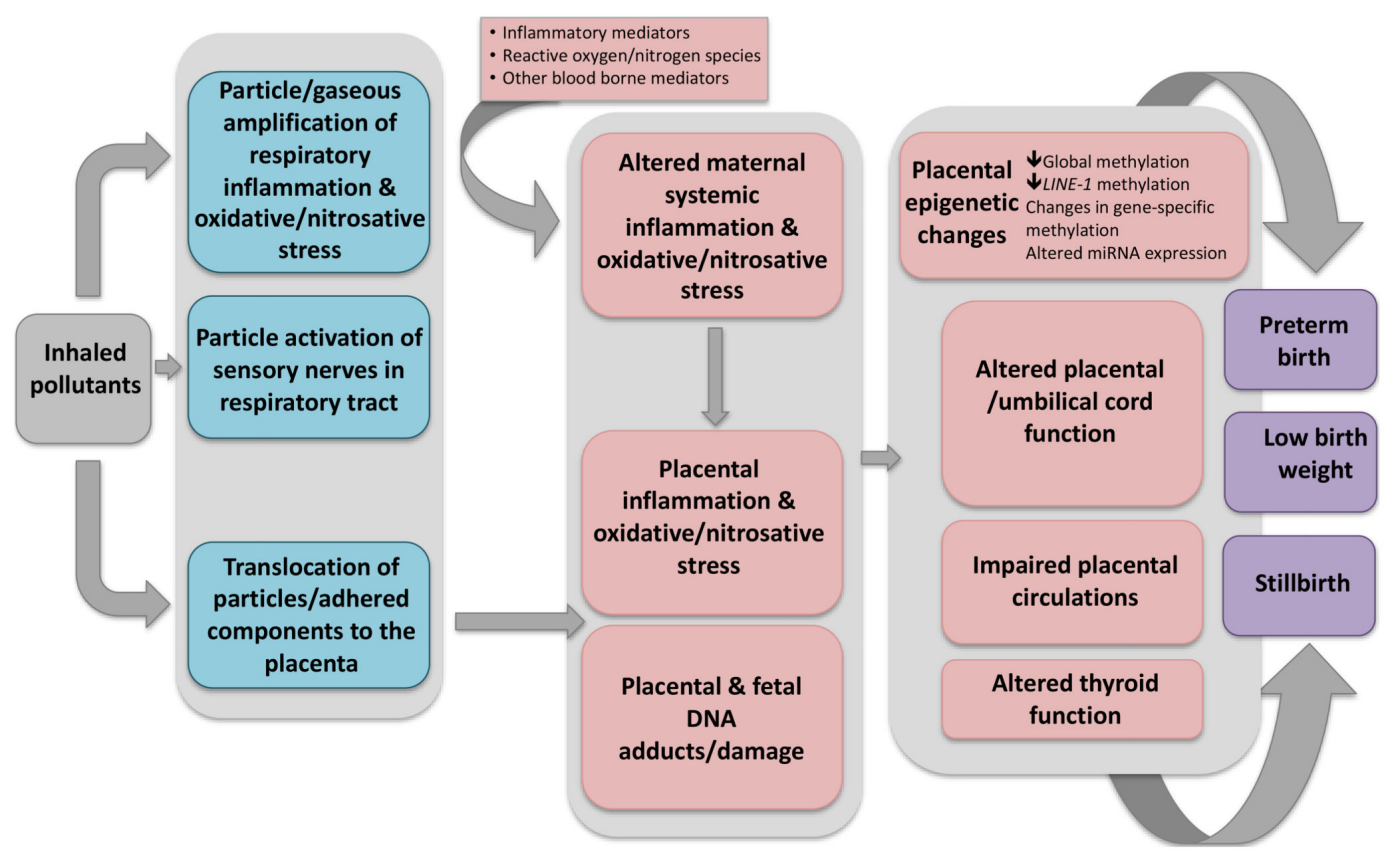
Biological mechanisms linking exposure to air pollutants to adverse birth outcomes

**Table 1:** Changes observed in human mechanistic studies in association with increased concentrations of ambient pollutants

Mechanism	Studied Changes	Result (in placenta and/or maternal/cord blood)
**Oxidative stress**	Oxidative DNA damage/tyrosine nitration	Increased with PM_2_._5_/PM_10_^51–54^
		Increased with NO_2_^53^
	mtDNA content	Decreased with PM_2_._5_/PM_10_^56–58^
		Decreased with NO_2_^59^
**Inflammation**	Intrauterine inflammation	Increased with PM2.5 ^79^
	Biomarkers (CRP, IL-1β, IL-6 & TNF-α)	Increased with PM_2.5_/PM_10_^77, 78, 80, 87^
		Increased with NO_2_^78^
		Increased with O_3_^77, 80^
**Epigenetic alterations**	Global DNA methylation	Decreased with PM_0.1_/ PM_2.5_/PM_10_/traffic ^97–100^
		Decreased with NO_2_^101^
		Decreased with O_3_^101^
	DNA methylation of HSD11B2	Increased with PM_2.5_^98^
	DNA methylation of H19	Decreased with PM_2.5_^104^
		Increased with NO_2_^104^

CRP= C-reactive protein; HSD11B211β-hydroxysteroid dehydrogenase 2 IL= interleukin; mtDNA = mitochondrial DNA; NO_2_= nitrogen dioxide; O_3_= ozone; PM_0.1_/ PM_2.5_/PM_10_ = particulate matter less than 0.1, 2.5 and 10 μm in diameter respectively; TNF-α= tumour necrosis factor-α

## Box 1 Key clinical points

- Epidemiological data provide varying degrees of evidence for associations between prenatal exposure to ambient air pollutants and adverse birth outcomes (suboptimal measures of fetal growth, preterm birth and stillbirth).
- Human studies show associations between concentrations of particulate and gaseous air pollution and increased placental vascular resistance to blood flow in the uterine circulation.
- Experimental evidence suggests exposure to air pollution during pregnancy may be associated with oxidative stress, inflammation and epigenetic alterations in placental tissue and/or maternal/cord blood as well as perturbations in the maternal fetal thyroid gland.
- In vitro work suggests PM can induce activation of stress pathways seen in the placenta in cases of FGR.

## Box 2 Future research

- Increased precision in quantifying (indoor and outdoor) air pollution exposure among pregnant individuals by employing reliable, low-cost portable devices/sensors and controlling the data for maternal tobacco smoke exposure by measuring cotinine levels in blood or urine.
- Use of multi-pollutant models to evaluate possible additive/synergistic effects of an overall mix of pollutants/identify independent effects of specific pollutants.
- Studies that investigate whether there is a critical exposure window during gestation for an effect of air pollution on birth outcomes.
- Greater research into mechanisms underlying the effects of ambient gaseous pollutants on adverse birth outcomes.
- More studies that link exposure to air pollutants and mechanistic endpoints with an adverse birth outcome and sex of the fetus.
- Continued use of cross-omic techniques to facilitate the identification of interacting genes/pathways that may underlie molecular mechanisms.
