# Supplementary material for: Ambient air pollution and adverse birth outcomes: A review of underlying mechanisms
Source: BJOG. Author manuscript; Available in PMC 2024 Apr 1. (PMC7615717; doi:10.1111/1471-0528.17727)
Supplement: Supplementary Material [file EMS191868-supplement-Supplementary_Material.pdf]

Table S1: Human studies linking maternal air pollutant concentrations and a given mechanistic endpoint with birth outcomes

Placental circulations					
**Reference**<br><br>**(Main manuscript citation number)**	**Population / location**	**Pollutant exposure**	**Placental endpoint**	**Birth outcome**	**Main findings**
Carvalho et al (2016)[1]<br><br>(38)	N=366<br>São Paulo, Brazil	NO_2 (mean µg/m^3)<br>T1: 42.9<br>T2: 39.2<br>T3: 37.2<br><br>O_3 (mean µg/m^3)<br>T1: 8.2<br>T2: 8.3<br>T3: 8.1	Umbilical artery PI<br><br>Middle cerebral artery PI<br><br>Uterine artery PI	Birth weight	O_3 in T2 was associated with higher umbilical artery PI values<br><br>O_3 in T3 was related to lower umbilical artery PI values<br><br>NO_2 did not influence umbilical artery PI values<br><br>*Birth outcome results*<br>• Birth weight had no association with NO_2 or O_3
Van den Hooven et al (2012)[2]<br><br>(42)	N=7801<br>Rotterdam, The Netherlands	PM_{10} (Mean WP µg/m^3): 30.3<br><br>NO_2 (Mean WP µg/m^3):	Umbilical artery PI<br><br>Uterine artery PI<br><br>Uterine artery notching	Placenta weight<br><br>Birth weight	PM_{10} & NO_2 averaged over WP associated with higher sFlt-1 (35.8%; 95% CI: 25.6, 45.9%; and 8.9%; 95% CI: 0.6, 17.3 per 10-µg/m3 increase in PM_{10} and NO_2, respectively) & lower PIGF–-16.3%; 95% CI: -21.9, -10.7 and -14.6%; 95% CI: –19.3,

		39.9	Cord blood PlGF<br><br>Cord blood sFlt-1		–10.0 per 10-µg/m^3 increase in PM_{10} & NO_2 respectively) levels in cord blood<br><br>PM_{10} & NO_2 not consistently associated with T2 or T3 placental resistance<br><br>*Birth outcome results*<br>• PM_{10} & NO_2 associated with lower placenta weight (-11.8 g; 95% CI: -20.9, -2.7 & -10.7 g; 95% CI: -19.0, -2.4, respectively per 10-µg/m^3 increase in the prior 2 months but not during the prior 2 weeks or over WP but not with placenta to birth weight ratio<br>• PM_{10} & NO_2 exposures during all three time periods associated with significant reductions in BW (–34.6; 95% CI: –66.3, –2.9; and –39.3; 95% CI: –69.1, –9.6 per 10-µg/m3 increase in PM_{10} and NO_2 during total pregnancy, respectively)

Oxidative stress

**Reference**<br><br>**(Main manuscript citation number)**	**Population/ location**	**Pollutant exposure**	**Assessment of oxidative stress**	**Birth outcome**	**Main findings**

Rossner et al (2011)[3]<br><br>(52)	N=891 Czech Republic<br><br>Prachatice: a district with low levels of air pollution<br><br>Teplice: at the time one of the most polluted districts of Czech Republic	$PM_{2.5}$ (pregnancy monthly means μg/m^3): 28.5-32.1	Placental 8-oxodG	IUGR<br><br>Term LBW	No difference in 8-oxodG between the districts, despite higher levels of air pollutants in the Teplice region<br><br>Using univariate analysis: $PM_{2.5}$ exposure during the second month of pregnancy was significantly associated with 8-oxodG levels in placental DNA.<br><br>*Birth outcome results*<br>• Borderline statistically significant elevated 8-oxodG levels found in IUGR group versus non-IUGR group (p=0.06)<br>• Slightly elevated levels of 8-oxodG found in term LBW group versus non-term LBW group ($p<0.05$)<br>• $PM_{2.5}$ concentrations not associated with IUGR but in 1st month of pregnancy was associated with risk of term LBW
Saenen et al (2016)[4]<br><br>53)	N=330 Genk, Belgium	$PM_{2.5}$ (Mean WP μg/m^3): 16.1<br><br>NO_2 (Mean WP μg/m^3): 20.5	Placental 3-NT	Birth weight<br><br>Birth length	Positive & significant correlation between placental 3-NT & WP exposure to $PM_{2.5}$ but not NO_2<br><br>For an IQR increment in $PM_{2.5}$ exposure over WP, placental 3-NT increased by 35.0% (95% CI: 13.9, 60.0; $P < 0.0006$).

					Birth outcome results<br>• No significant associations between placental 3-NT & birth weight or birth length
Grevendonk et al (2016)[5]<br><br>(54)	N=224-293<br>Genk, Belgium	$PM_{2.5}$ (Medium WP µg/m^3): 21.4<br><br>PM_{10} (Medium WP µg/m^3): 16.6	Maternal blood mitochondrial 8-OHdG	Birth weight<br><br>SGA	PM_{10} exposure during WP was positively associated with mitochondrial 8-OHdG in maternal blood (18.3, 95 % CI: 5.6 to 33.4 %, p = 0.004 for an IQR increment in PM_{10} exposure)<br><br>$PM_{2.5}$ exposure during WP was positively associated with mitochondrial 8-OHdG in maternal blood (13.9, 95 % CI: 0.4 to 29.4 %, p = 0.04 for an IQR increment in $PM_{2.5}$ exposure)<br><br>No significant association between cord blood 8-OHdG levels & PM_{10} or $PM_{2.5}$ exposure during WP<br><br>PM_{10} (but not $PM_{2.5}$) exposure during T1 & T2 was positively associated with mitochondrial 8-OHdG in cord blood (23.0, 95 % CI: 5.9 to 42.8 %, p = 0.007 & 16.6%, 95% CI: 1.8 to 33.5%, p=0.03 respectively for an IQR increment in PM_{10} exposure)<br><br>*Birth outcome results*

					• Neither PM exposure nor 8-OHdG levels in cord & maternal blood were associated with birth weight or SGA
Clemente et al (2016)[6]<br><br>(59)	Belgium (N=550) & Spain (N=376)	NO_2 (mean WP μg/m^3)<br><br>Belgium: 21.1<br>Spain: 25.2<br>Belgium & Spain: 22.7	Placental mtDNA content	Birth weight	10 μg/m^3 increment in average NO_2 exposure during pregnancy was associated with a 4.9% decrease in placental mtDNA content<br><br>*Birth outcome results*<br>• 10 μg/m^3 increment in average NO_2 exposure during pregnancy was associated with a 48g decrease in BW<br>• Placental mtDNA content was positively & significantly associated with birth weight in both cohorts & in the pooled analysis<br>• IQR increase in mtDNA content was significantly associated with a 66g increase in birth weight in boys but there was no significant difference in girls
Lavigne et al (2018)[7]<br><br>(64)	N=196,171 Ontario, Canada	$PM_{2.5}$ (Mean μg/m^3)<br>T1: 9.0<br>T2: 9.1<br>T3: 9.0<br>WP: 9.0	-	PTB<br><br>Term LBW<br><br>Birth weight	*Birth outcome results*<br>• IQR increase (2.6 μg/m^3) in T1 $PM_{2.5}$ positively associated with term LBW among women in the highest quartile of OP^{GSH} (OR=1:28; 95% CI: 1.10, 1.48), but not the lowest quartile (OR = 0:99; 95% CI: 0.87, 1.14; p-interaction = 0:03)

					• $PM_{2.5}$ on day of delivery associated with PTB among women in the highest quartile of GSH-related oxidative potential (HR= 1:02; 95% CI: 1.01, 1.04), but not the lowest quartile (HR = 0:97; 95% CI: 0.95, 1.00; p-interaction = 0:04)<br>• Between-city differences in OP^{AA} did not significantly modify associations with $PM_{2.5}$
Borlaza et al (2022)[8]<br><br>(65)	N=405<br>Grenoble, France	$PM_{2.5}$ (Mean μg/m^3)<br>≤37 weeks: 15.4<br>38-39 weeks: 13.4<br>39-42 weeks: 14.4	-	Birth weight<br><br>Birth height<br><br>Birth head circumference	*Birth outcome results*<br>No evidence of interaction between $PM_{2.5}$ mass concentration and OP^{DTT} or OP^{AA} for any of the birth outcomes considered
Suh et al (2008)[9]<br><br>(66)	N=235<br>Seoul, Korea	PM_{10} (Medium μg/m^3)<br>T1: 61.12<br>T2: 59.05<br>T3: 54.64	Maternal blood +/- GSTM1 null genotype	PTB	*Birth outcome results*<br>• Risk of PTB conferred by exposure to high levels of PM_{10} (i.e.≥75th percentile) was significant in T3 (OR = 2.33, 95% CI = 1.33–4.80, p = 0.022), but not in T1 or T2 (p > 0.05).<br>• Risk of PTB was associated with the GSTM1 null genotype but not GSTM1 positive genotype<br>• Exposure to high levels (≥75th

					percentile) of PM_{10} during T3 in the presence of the GSTM1 null genotype is significantly associated with risk of PTB
Zhao et al (2021)[10]<br><br>(67)	N=436<br>Taiyuan, China	Chinese AQI	Maternal blood SNPs in:<br>GPx4<br>GLRX<br>EGF<br>IL-1α	PTB	*Birth outcome results*<br>• Higher levels of maternal AQI exposure associated with a higher risk of PTB among women who carried polymorphisms in genes for GPX4, GLRX, EGF & IL-1α alpha<br><br>• After adjustment for multiple comparisons, only *GPX4*-rs376102 and AQI interaction remained statistically significant

Inflammation

**Reference**<br><br>**(Main manuscript citation number)**	**Population Location & number**	**Pollutant – assessment & concentration**	**Inflammatory biomarker**	**Birth outcome assessed**	**Main findings**
Friedman et al (2021)[11]<br><br>(80)	N=515<br>Denver, Colorado	$PM_{2.5}$ (Median WP μg/m^3): 7.51<br><br>O_3 (Median WP	Maternal blood CRP, IL-6, TNFα	Birth weight<br><br>Gestational age	*Maternal CRP*<br>$PM_{2.5}$ & O_3: No significant associations

		ppb): 44			*Maternal IL-6*<br>$PM_{2.5}$: Positive associations with T1& prior 30-day concentrations<br>O_3: Negative associations with prior 30- and 7-day concentrations & positive associations with T1 concentrations<br><br>*Maternal TNFα*<br>$PM_{2.5}$: Positive associations with T1, T2 & prior 30-day concentrations<br>O_3: Positive associations with T1 & T2 concentrations<br><br>*Cord blood CRP, IL-6, TNFα*<br>Patterns inconsistent for associations between $PM_{2.5}$ and O_3<br><br>*Birth outcome results*<br>• Maternal CRP positively associated with infant gestational age at birth (β; 1.30, 95% CI; 0.40–2.21); no associations between maternal CRP, IL-6 & TNFα & birth weight
Epigenetic alterations					

**Reference**<br><br>**(Main manuscript citation number)**	**Population / location**	**Pollutant exposure**	**Epigenetic endpoint**	**Birth outcome assessed**	**Main findings**
Liu et al (2019)[12]<br><br>(97)	N=4278 Guangzhou, China	$PM_{2.5}$ (Mean WP µg/m^3): 32.07<br><br>PM_{10} (Mean WP µg/m^3): 48.67<br><br>PM_1 (Mean WP µg/m^3): 27.93	Maternal & cord blood DNA methylation of LINE-1	PTB	Significant negative associations of PM_{10}, $PM_{2.5}$ & PM_1 concentrations with cord blood LINE-1 methylation levels<br><br>Negative association between PM_1 concentration & maternal LINE-1 methylation level<br><br>*Birth outcome results*<br>• Risk of PTB positively associated with $PM_{2.5}$ and PM_1 during gestational weeks 12-20<br>• Risk of PTB negatively associated with maternal blood LINE-1 methylation levels & positively correlated with cord blood LINE-1 methylation levels but findings not significant
Cai et al (2017)[13]<br><br>(98)	N=181 Shanghai, China	PM_{10} (Median µg/m^3)<br>WP: 63.51<br>T1: 62.74<br>T2: 68.07<br>T3: 65.29	Placental DNA methylation of LINE1, HSD11B2 & NR3C1	Fetal growth restriction	Placental LINE-1 DNA methylation reversely associated with T1 PM_{10} concentrations<br><br>Placental HSD11B2 DNA methylation was associated with T1 & T2 PM_{10}

					Birth outcome results<br>• Above associations were much more evident in FGR newborns
Kingsley et al (2016)[14]<br><br>(100)	N=471<br>Rhode Island, USA	Residential proximity to roadway (defined as living ≤ 150 m from a primary highway or primary road or ≤ 50 m from a secondary road)	Placental DNA methylation of repetitive elements (LINE-1 and AluYb8)	Birth weight<br><br>SGA	Living close to major roadway associated with:<br><br>175.9 g (95% CI: -319.4, -32.5; p=0.016) lower birth weight<br><br>Lower mean placental LINE-1 methylation levels<br><br>Differential methylation of 7 CpG sites (in n=215 sub-cohort)<br><br>*Birth outcome results*<br>• Additional adjustment for placental methylation did not attenuate the association between roadway proximity & birth weight
He et al (2018)[15]<br><br>(104)	N=527<br>Zhengzhou, China	PM_{10} (Mean μg/m^3)<br>WP: 106.66<br>T1: 104.88<br>T2: 107.57<br>T3: 107.27	Maternal & cord blood DNA methylation of H19 in promoter region & DMR	Birth weight<br><br>Birth length	PM_{10} & NO_2 concentrations during WP associated with lower H19 DMR methylation & higher H19 methylation in cord blood respectively<br><br>*Birth outcome results*<br>• Statistically significant associations

		NO_2 (Mean µg/m^3)<br>WP: 44.16<br>T1: 44.71<br>T2: 44.65<br>T3: 43.10			between NO_2 & PM_{10} exposures on birth weight & length but these varied by different trimesters & were sometimes positively associated & sometimes negatively associated<br>• No mediating effects of H19/DMR methylation in maternal or cord blood were found in the relationship between air pollutants exposure during pregnancy and birth sizes
Vos et al (2020)[16]<br><br>(105)	N=40<br>Genk, Belgium	High ($PM_{2.5}$: 16.0 ± 1.4 µg/m^3) versus low ($PM_{2.5}$: 10.6 ±1.7 µg/m^3)	Placental mtDNA methylation in: D-loop & LDLR2 regions	Birth weight	Absolute D-loop methylation levels higher for mothers highly exposed to air pollutants (+0.47%, 95% CI: 0.20% to 0.73%)<br><br>D-loop methylation levels correlated with placental mtDNA content (r = −0.40, p = 0.002)<br><br>*Birth outcome results*<br>• D-loop methylation levels associated with birth weight (−106.98 g, 95% CI: −209.60 g to −4.36 g for an IQR increase in D-loop methylation)
Endocrine disruption					

Reference<br><br>(Main manuscript citation number)	Population Location & number	Pollutant – assessment & concentration	Biomarker	Birth outcome assessed	Main findings
Janssen et al (2017)[17]<br><br>(111)	N=499/431 Genk, Belgium	$PM_{2.5}$ (Mean T3 μg/m^3): 16	Maternal & cord blood FT_3, FT_4 & TSH at delivery	Birth weight	IQR increment (8.2 μg/m3) in T3 $PM_{2.5}$ inversely associated with:<br>• Cord blood TSH levels (–11.6%; 95% CI: –21.8, –0.1)<br>• FT4/FT3 ratio (–62.7%; 95% CI: –91.6, –33.8)<br><br>*Birth outcome results*<br>• 10th–90th percentile decrease in cord blood FT4 levels associated with a 56 g decrease in mean birth weight (95% CI: –90, –23)
Wang et al (2019) [18]<br><br>(112)	N=433 Nanjing, China	$PM_{2.5}$ (Mean T2 μg/m^3):	Maternal serum FT_4, TSH, TPOAb during T2<br><br>Neonatal TSH within 72 hours after birth	Birth weight	Higher exposure to $PM_{2.5}$ decreased maternal FT_4 level ($p < 0.05$)<br><br>*Birth outcome results*<br>• Birth weight Z-score was decreased ($p < 0.05$) by higher exposure to maternal $PM_{2.5}$<br><br>• Mediation analysis clarified that maternal FT_4 levels explained

					15.9%, of the association of maternal $PM_{2.5}$ exposure with birth weight Z-score, ($p < 0.05$)
Metabolomics					
**Reference**<br><br>**(Main manuscript citation number)**	**Population Location & number**	**Pollutant – assessment & concentration**	**Birth outcome assessed**	**Approach/sample**<br><br>**Metabolites identified**	**Pathway enrichment**
Laine et al (2020)[19]<br><br>(125)	N=498<br>4 cohorts in: Belgium, Spain, Italy, Greece	Multi-pollutant modeling:<br><br>5-pollutant mixture of $PM_{2.5}$, PM_{10}, NO_2, NO_x & UFP in all cohorts except ENVIRONAGE & 5-pollutant mixture of $PM_{2.5}$, PM_{10}, NO_2, OP_{AA}, & OP_{GSH} in all cohorts except Rhea	Birth weight	Cord blood<br><br>4712 features through untargeted approach<br><br>665 features through cross-omics (metabolome & inflammatory proteome)	Top 10:<br>Aspartate & asparagine metabolism<br>Arginine & proline metabolism, Tyrosine metabolism,<br>Urea cycle/amino group metabolism,<br>Glycerophospholipid metabolism,<br>Fatty acid activation,<br>Tryptophan metabolism,<br>Arachidonic acid metabolism,<br>Vitamin A metabolism<br>Prostaglandin formation from arachidonate<br><br>*Birth outcome results*<br>• Negative association with birth weight z-scores & exposure to mixtures of air

		Mean concentrations (year before birth)<br><br>$PM_{2.5}$: 19 μg/m^3<br>PM_{10}: 35 μ g/m^3<br>NO_2: 30 μg/m^3<br>NO_x: 73 μ g/m^3<br>OP^{AA}: 62 OP/m^3<br>OP^{GSH}: 5 OP/m^3<br>UFP: 13393 n/cm^3			pollutants: Up to a 96 g decrease in birth weight, comparing the 75th percentile to the median level of exposure to the air pollutant mixture could occur<br><br>• Shifts in birth weight z-scores from prenatal exposure to $PM_{2.5}$ PM_{10} , & NO_2 mediated by molecular mechanisms, represented by cross-omics scores<br><br>• IL-17 & epidermal growth factor identified as important responses underlying air pollution-associated shifts in birth weight.

Abbreviations: 3-NT: 3-nitrotyrosine; 8-OHdG: 8-hydroxy-2'-deoxyguanosine; 8-oxodG: 8-oxo-7,8-dihydro-2 -deoxyguanosine; AQI: air quality index; CI: confidence interval; CRP: C-reactive protein; DMR: differentially methylated regions; EGF: endothelial growth factor; FST: thyroid stimulating hormone; FT_3: free triiodothyronine; FT_4: free thyroxine; GLRX: glutaredoxin; GPX4: glutathione peroxidase 4; GSTM1: glutathione S-Transferase Mu 1; HR: hazard ratio; IL: interleukin; IUGR: intrauterine growth restriction; IQR: interquartile range; LBW: low birth weight; LINE: long interspersed nucleotide elements; mtDNA: mitochondrial DNA; NO_2: nitrogen dioxide; NO_x: oxides of nitrogen; O_3: ozone; OP^{AA}: ascorbate-related oxidative potential; OP^{DTT}: dithiothreitol-related OP; OP^{GSH}: glutathione-related oxidative potential; OR: odds ratio; PI: pulsatility index; PIGF: placental growth factor; PM_1: particulate matter < 0 μm in diameter; PM_{10}: particulate matter < 10 μm in diameter; $PM_{2.5}$: particular matter < 2.5 μm in diameter; PTB: preterm birth; sFlt-1: soluble fms-like tyrosine kinase 1; SGA: small for gestational age; T: trimester; TNF-α: tumor necrosis factor alpha; TPOAb: thyroid peroxidase antibody; TSH: thyroid stimulating hormone; UFP: ultrafine particles; WP: whole pregnancy

References

1. Carvalho MA, Bernardes LS, Hettfleisch K, Pastro LD, Vieira SE, Saldiva SR, et al. Associations of maternal personal exposure to air pollution on fetal weight and fetoplacental Doppler: A prospective cohort study. Reprod Toxicol. 2016 Jul;62:9-17.

2. van den Hooven EH, Pierik FH, de Kluizenaar Y, Hofman A, van Ratingen SW, Zandveld PY, et al. Air pollution exposure and markers of placental growth and function: the generation R study. Environ Health Perspect. 2012 Dec;120(12):1753-9.

3. Rossner P, Jr., Tabashidze N, Dostal M, Novakova Z, Chvatalova I, Spatova M, et al. Genetic, biochemical, and environmental factors associated with pregnancy outcomes in newborns from the Czech Republic. Environ Health Perspect. 2011 Feb;119(2):265-71.

4. Saenen ND, Vrijens K, Janssen BG, Madhloum N, Peusens M, Gyselaers W, et al. Placental Nitrosative Stress and Exposure to Ambient Air Pollution During Gestation: A Population Study. Am J Epidemiol. 2016 Sep 15;184(6):442-9.

5. Grevendonk L, Janssen BG, Vanpoucke C, Lefebvre W, Hoxha M, Bollati V, et al. Mitochondrial oxidative DNA damage and exposure to particulate air pollution in mother-newborn pairs. Environ Health. 2016 Jan 20;15:10.

6. Clemente DB, Casas M, Vilahur N, Begiristain H, Bustamante M, Carsin AE, et al. Prenatal Ambient Air Pollution, Placental Mitochondrial DNA Content, and Birth Weight in the INMA (Spain) and ENVIRONAGE (Belgium) Birth Cohorts. Environ Health Perspect. 2016 May;124(5):659-65.

7. Lavigne E, Burnett RT, Stieb DM, Evans GJ, Godri Pollitt KJ, Chen H, et al. Fine Particulate Air Pollution and Adverse Birth Outcomes: Effect Modification by Regional Nonvolatile Oxidative Potential. Environ Health Perspect. 2018 Jul;126(7):077012.

8. Borlaza LJS, Uzu G, Ouidir M, Lyon-Caen S, Marsal A, Weber S, et al. Personal exposure to PM2.5 oxidative potential and its association to birth outcomes. Journal of Exposure Science & Environmental Epidemiology. 2022.

9. Suh YJ, Ha EH, Park H, Kim YJ, Kim H, Hong YC. GSTM1 polymorphism along with PM10 exposure contributes to the risk of preterm delivery. Mutat Res. 2008 Oct 30;656(1-2):62-7.

10. Zhao N, Wu W, Feng Y, Yang F, Han T, Guo M, et al. Polymorphisms in oxidative stress, metabolic detoxification, and immune function genes, maternal exposure to ambient air pollution, and risk of preterm birth in Taiyuan, China. Environmental Research. 2021;194:110659.

11. Friedman C, Dabelea D, Thomas DSK, Peel JL, Adgate JL, Magzamen S, et al. Exposure to ambient air pollution during pregnancy and inflammatory biomarkers in maternal and umbilical cord blood: The Healthy Start study. Environ Res. 2021 Apr 12:111165.

12. Liu X, Ye Y, Chen Y, Li X, Feng B, Cao G, et al. Effects of prenatal exposure to air particulate matter on the risk of preterm birth and roles of maternal and cord blood LINE-1 methylation: A birth cohort study in Guangzhou, China. Environ Int. 2019 Dec;133(Pt A):105177.

13. Cai J, Zhao Y, Liu P, Xia B, Zhu Q, Wang X, et al. Exposure to particulate air pollution during early pregnancy is associated with placental DNA methylation. Sci Total Environ. 2017 Dec 31;607-608:1103-8.

14. Kingsley SL, Eliot MN, Whitsel EA, Huang YT, Kelsey KT, Marsit CJ, et al. Maternal residential proximity to major roadways, birth weight, and placental DNA methylation. Environ Int. 2016 Jul-Aug;92-93:43-9.

15. He T, Zhu J, Wang J, Ren X, Cheng G, Liu X, et al. Ambient air pollution, H19/DMR methylation in cord blood and newborn size: A pilot study in Zhengzhou City, China. Chemosphere. 2018 Aug 30;212:863-71.

16. Vos S, Nawrot TS, Martens DS, Byun H-M, Janssen BG. Mitochondrial DNA methylation in placental tissue: a proof of concept study by means of prenatal environmental stressors. Epigenetics. 2020:1-11.

17. Janssen BG, Saenen ND, Roels HA, Madhloum N, Gyselaers W, Lefebvre W, et al. Fetal Thyroid Function, Birth Weight, and in Utero Exposure to Fine Particle Air Pollution: A Birth Cohort Study. Environ Health Perspect. 2017 Apr;125(4):699-705.

18. Wang X, Liu C, Zhang M, Han Y, Aase H, Villanger GD, et al. Evaluation of Maternal Exposure to PM(2.5) and Its Components on Maternal and Neonatal Thyroid Function and Birth Weight: A Cohort Study. Thyroid : official journal of the American Thyroid Association. 2019 Aug;29(8):1147-57.

19. Laine JE, Bodinier B, Robinson O, Plusquin M, Scalbert A, Keski-Rahkonen P, et al. Prenatal Exposure to Multiple Air Pollutants, Mediating Molecular Mechanisms, and Shifts in Birthweight. Environmental Science & Technology. 2020.
